# Phosphite Protects *Fagus sylvatica* Seedlings towards *Phytophthora plurivora* via Local Toxicity, Priming and Facilitation of Pathogen Recognition

**DOI:** 10.1371/journal.pone.0087860

**Published:** 2014-01-28

**Authors:** Ronaldo J. D. Dalio, Frank Fleischmann, Martina Humez, Wolfgang Osswald

**Affiliations:** Section Pathology of Woody Plants, Technische Universität München, Freising, Germany; University of Nebraska-Lincoln, United States of America

## Abstract

*Phytophthora plurivora* causes severe damage on *Fagus sylvatica* and is responsible for the extensive decline of European Beech throughout Europe. Unfortunately, no effective treatment against this disease is available. Phosphite (Phi) is known to protect plants against *Phytophthora* species; however, its mode of action towards *P. plurivora* is still unknown. To discover the effect of Phi on root infection, leaves were sprayed with Phi and roots were subsequently inoculated with *P. plurivora* zoospores. Seedling physiology, defense responses, colonization of root tissue by the pathogen and mortality were monitored. Additionally the Phi concentration in roots was quantified. Finally, the effect of Phi on mycelial growth and zoospore formation was recorded. Phi treatment was remarkably efficient in protecting beech against *P. plurivora*; all Phi treated plants survived infection. Phi treated and infected seedlings showed a strong up-regulation of several defense genes in jasmonate, salicylic acid and ethylene pathways. Moreover, all physiological parameters measured were comparable to control plants. The local Phi concentration detected in roots was high enough to inhibit pathogen growth. Phi treatment alone did not harm seedling physiology or induce defense responses. The up-regulation of defense genes could be explained either by priming or by facilitation of pathogen recognition of the host.

## Introduction

Protecting plants from *Phytophthora* species is still a challenge. In the last decade a large number of new species have been described beyond agriculture which are known to cause enormous economic and environmental losses [1], thus driving the attention of the scientific community towards the genus *Phytophthora* [2].

*Phytophthora plurivora* is a hemibiotrophic root pathogen with worldwide distribution, attacking several plant species, in particular, *Fagus sylvatica* (European beech) [3], [4]. *F. sylvatica* is a dominant species in most of the European forests, with great economic value. The decline of *F. sylvatica* in European forests was associated with the interaction with *P. plurivora* (formerly called *P. citricola*) and climatic extremes [5].

During evolution plants have developed defense mechanisms that enable them to recognize invaders and to avoid colonization by most pathogenic microorganisms [6]. Thus resistance is the rule and susceptibility the exception. The defense mechanisms in plants consist of a two-layered immune-system [7]. The first layer depends on recognition of PAMPs (pathogen associated molecular patterns) by pattern-recognition receptors (PRRs) localized at plasma membrane structures, resulting in PAMP triggered immunity (PTI). Pathogens try to circumvent plant PTI and therefore evolved effectors to suppress PAMP-triggered defense that leads to effector triggered susceptibility (ETS). In order to defend itself against these pathogen-derived effectors, plants have developed specific receptor (R) proteins as the second layer of defense. These R-proteins recognize effector proteins of the invader directly or indirectly leading to effector triggered immunity (ETI) [8]. However, successful pathogens, including *Phytophthora* species, have the ability to subvert these defense mechanisms either by avoiding their recognition or by reprogramming host metabolism [9], [10]. The *P. plurivora* - *F. sylvatica* interaction is characterized by a “striking lack of defense gene induction” and it was concluded that *P. plurivora* possibly escapes the main plant-recognition systems [11]. Thus, supporting the recognition of the invader by the host might be a key-factor in managing *Phytophthora* diseases.

Plant recognition of invaders commonly causes a fast flux of ions, the accumulation of reactive oxygen species (ROS), the activation of MAP kinases signaling cascades as well as specific gene expression and finally the activation of defense pathways [12]. Induced downstream responses rely in most cases on a network of cross-communication between signaling pathways mediated by salicylic acid (SA), jasmonate (JA) or ethylene (ET) [13].

Unfortunately, *Phytophthora* pathogens cannot be controlled with well-known fungicides, because as Oomycetes they do not synthesize chitin and ergosterol. However, many investigations have shown that different salts of the phosphorous acid, the phosphites, are effective in the control of *Phytophthora* pathogens. Bark injection of phosphite successfully controlled sudden oak death of coast life oaks caused by *P. ramorum* [14]. It was also proved that phosphite treatment of *Arabidopsis thaliana* leaves triggered the release of superoxide, caused localized cell death and enhanced accumulation of phenolics around infected cells [15]. In consequence growth of *P. palmivora* was restricted and the production of sporangia was inhibited. In the presence of the superoxide quencher Mn(II)-desferal, there was no longer hypersensitive cell death and the pathogen was able to grow in phosphite-treated plants. These data prove that inhibition of the pathogen was due to superoxide release rather than a direct effect of the chemical. Phosphite treatment was recently shown to prime *Arabidopsis thaliana* for defense responses of the salicylic acid (SA) and jasmonic acid/ethylene (JA/ET) pathways [16]. Thus, phosphite treated plants showed a significant reduction in lesion size after infection with *P. cinnamomi*.

Besides interfering with defense pathways of host plants, phosphite was shown to interact directly with *Phytophthora* pathogens. The chemical caused hyphal distortion and lyses of cell walls in parallel with a down-regulation of many genes encoding proteins involved in cell wall synthesis and cytoskeleton functioning [17]. It was also demonstrated that oospore production, sporangia formation and mycelial growth of *P. cinnamomi* and *P. citricola* were significantly inhibited by either phosphorous acid or fosetyl-Al [18]. It was concluded that phosphite acts in a dual way: when phosphite concentrations are low, the chemical induced host defense enzymes such as 4-coumarate coenzyme A ligase (4-CL) and cinnamyl alcohol dehydrogenase (CAD). However, at high phosphite concentrations, the chemical acted directly on *P. cinnamomi* and inhibited its growth [19].

Extensive field studies have proved that phosphite treatment of *Eucalyptus* and *Banksia* species is a practical option to control *P. cinnamomi* root infection over several years [20] [21]. However, glasshouse studies showed that phosphite treatment reduced but did not prevent the production of viable zoospores on infected trees [22]. Thus, the authors concluded that phosphite application will lower the amount of infection by *Phytophthora* species, but may not remove the risk of *Phytophthora* spreading from already infected trees.

The aim of this study was to elucidate the mechanisms of potassium phosphite on plant physiology and gene regulation of treated beech saplings in order to understand the protective effect of this chemical on the highly susceptible interaction between *F. sylvatica* and *P. plurivora*.

## Results

### *In vitro* inhibition of mycelial growth and zoospore inhibition of *P. plurivora* by phosphite

**Figure 1:**
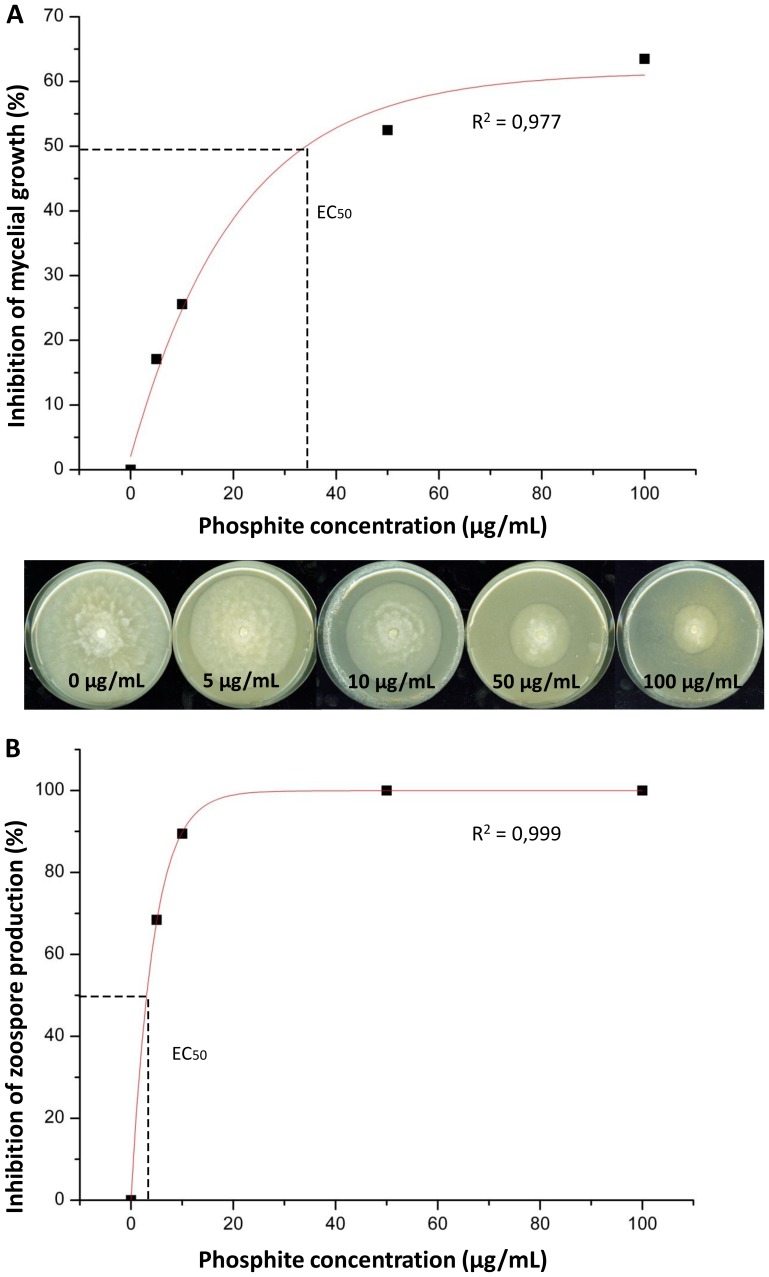
Effect of phosphite on *in vitro* growth and zoospore production of *P. plurivora*. (a) Inhibition of *P. plurivora* mycelial growth using different phosphite concentrations. (b) *P. plurivora* cultures in Petri dishes illustrating the inhibition of mycelial radial growth with increasing phosphite concentrations. The mycelial colonies were six days old. (c) Inhibition of *P. plurivora* zoospore production at different phosphite concentrations. EC_50_ shows the concentration that inhibits growth or zoospore production to 50%. Trend-lines were fitted using a logarithmic function. These assays were repeated three times showing similar results. n = 5 for each assay.

Inhibition of mycelial growth started at 5 µg/mL and reached 65% inhibition with 100 µg/mL of phosphite (Figure 1a and 1b). The EC_50_ value was calculated as 34 µg/mL. The number of zoospores released was significantly lower for all phosphite treatments (Figure 1c). The EC_50_ value for zoospore inhibition was determined as 2.9 µg/mL, about ten times lower than the inhibition of mycelial growth.

### Physiological plant responses to phosphite treatment and infection

**Figure 2:**
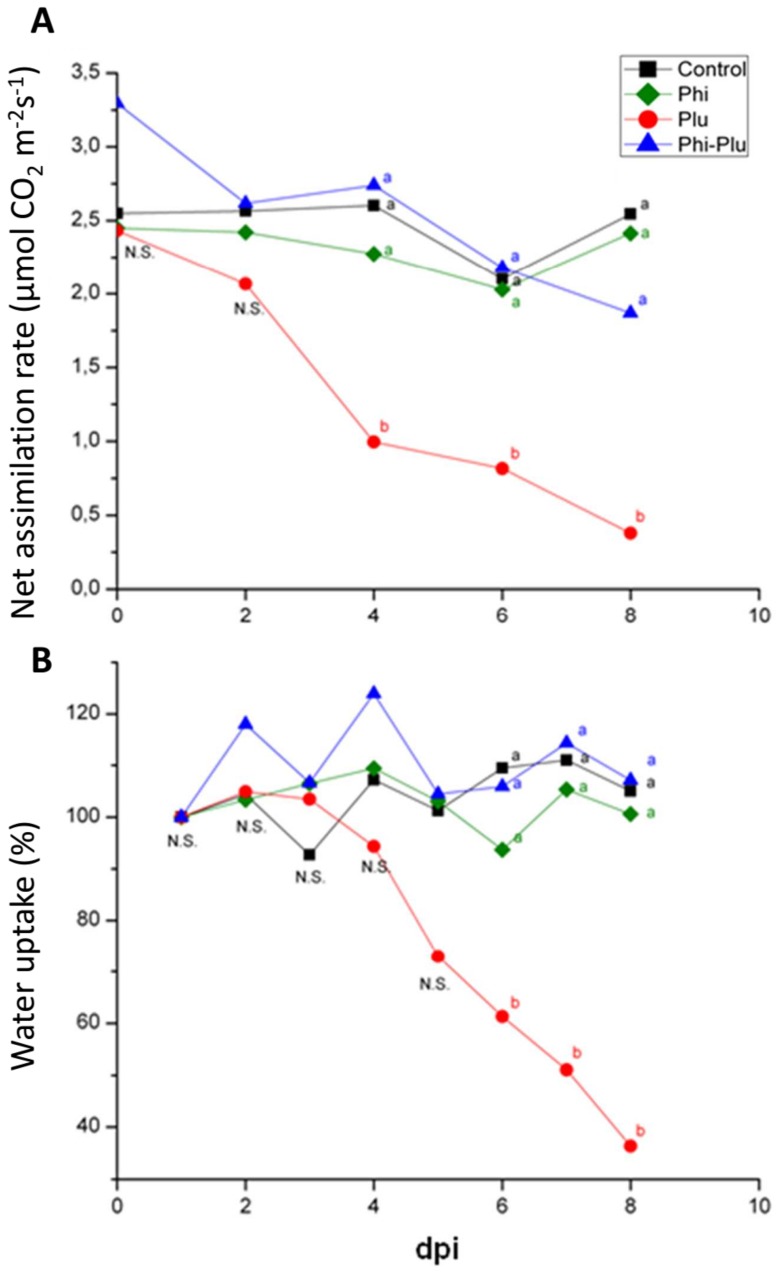
Effect of phosphite treatment and *P. plurivora* infection on net CO_2_ assimilation rates (µmol CO_2_ m^−2^s^−1^) (a) and water uptake (b) over ten days. Water uptake was calculated as percent of g/cm^2^ leaf surface of four months-old beech saplings. Treatments: Control: not infected and not phosphite treated plants; Phi: phosphite treatment; Plu: roots inoculated with zoospores of *P. plurivora*; Phi-Plu: foliar application of 0.5% phosphite on plants prior to inoculation with zoospores of *P. plurivora*. The experiment was repeated three times showing similar results. N = 6 plants per treatment. dpi- days post inoculation. Different letters at the same time points show statistical differences (P≤0.05), N.S. =  Not-significant.

Phosphite treatment did not show any beneficial or adverse effect on physiological parameters of not-inoculated plants. No significant differences were found for net CO_2_ assimilation, water uptake, Jmax or Vcmax in comparison with control plants (Figure 2 a, b, and Table 1).

**Table 1:** Rubisco activity (Vcmax) and maximum rate of electron transport (Jmax) data fitted from A/ci curves of *F. sylvatica* seedlings at 8 days post inoculation.

	A/ci curves fitted parameters	
Treatment	V_cmax_ [µmol CO_2_ m^−2^s^−1^]	J_max_ [µmol e^−^ m^−2^s^−1^]
Control	12.17 a*	24.10 a
Phi	10.22 a	19.46 ab
Plu	4.99 b	19.46 ab
Phi-Plu	10.42 a	26.01 a

* Same letters within a column represent no statistical differences, Tukey test (P≤0,05).

However, *P. plurivora* infection strongly affected plant physiology of not phosphite –treated saplings. CO_2_ assimilation rates and water uptake strongly decreased during the experiment, reaching almost zero values at 10 dpi (Figure 2 a, b). VcMax and Jmax were also affected, showing significantly lower values as compared to control plants (Table 1).

Phosphite treatment converted the susceptible interaction between *F. sylvatica* and *P. plurivora* into a resistant one. All physiological parameters analyzed did not differ to control plants (Figure 2a, b; and Table 1).

### Symptoms and mortality of plants

**Figure 3:**
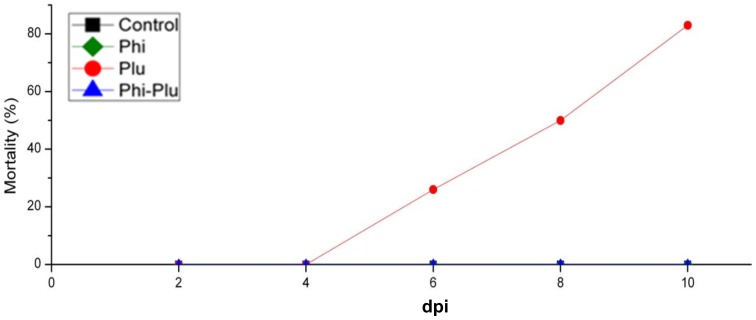
Effect of phosphite treatment and *P. plurivora* infection on plant mortality over time. Treatments: Con (black square): not phosphite treated and not inoculated control plants; Phi (green square): plants sprayed with phosphite (0.5%) on leaves until run off; Plu (red circle): roots infected with *P. plurivora*; Phi-Plu (blue triangle): plants sprayed with phosphite (0.5%) on leaves until run off four days prior to infection with *P. plurivora*. n = 6 plants per treatment. The experiment was repeated three times showing similar results. dpi: days post inoculation.

No symptoms or mortality were recorded for not-inoculated control plants and those treated with phosphite (Figure 3). Wilting symptoms were recorded for inoculated plants after 4 dpi. At the end of the experiment, 83% of the infected plants had died. Plants treated with Phi and inoculated with *P. plurivora* showed no symptoms and no mortality during the whole experiment (Figure 3).

### Quantification of phosphite and *P. plurivora* in roots

**Figure 4:**
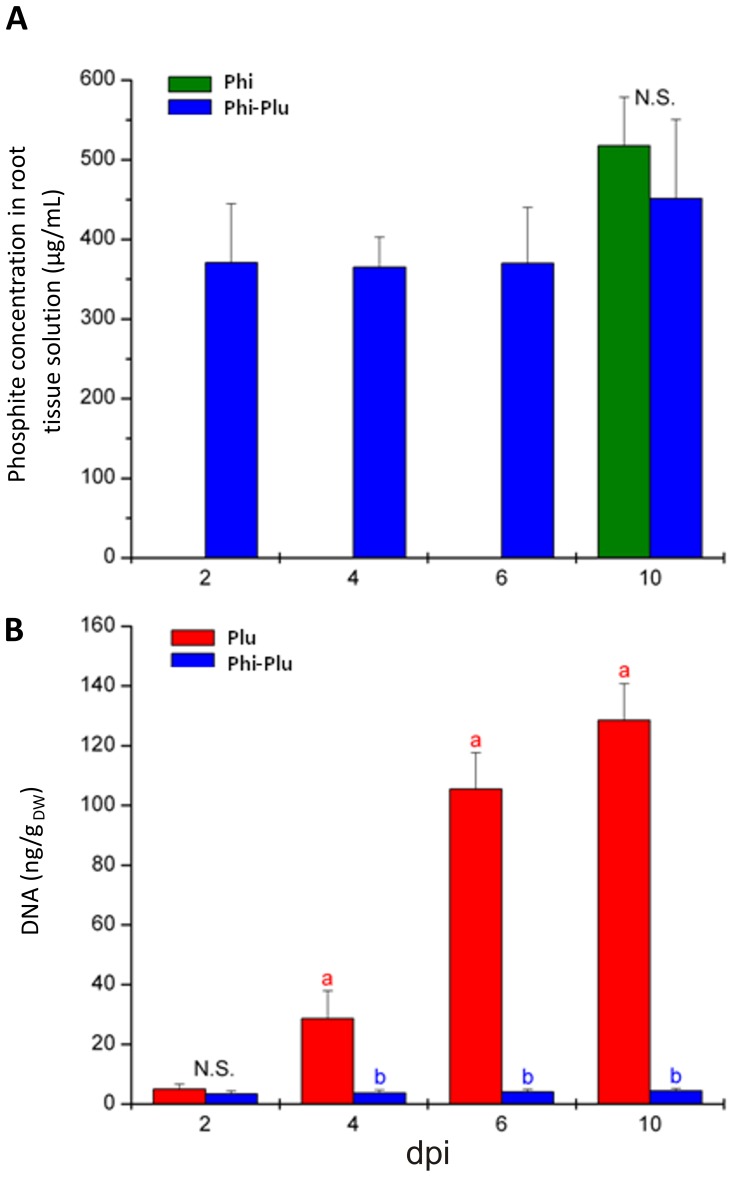
Concentrations of phosphite and *P. plurivora* DNA along time. (a) Phosphite concentrations of root tissue. (b) qPCR analysis of *P. plurivora* DNA along time (ng/gDW). Red bars: Plants infected with *P. plurivora*; blue bars: Plants infected with *P. plurivora* and treated with phosphite (0.5%). Green bars: Plants treated with Phi. n = 6 plants per treatment. The experiments were repeated 3 times showing similar results. Dpi: days post inoculation. Different letters at the same time points show statistical differences (P≤0.05), N.S. =  Not-significant.

The concentration of phosphite in infected roots ranged from 370 to 510 µg/mL during the experiment (Figure 4a). These concentrations are about ten times higher than those necessary to inhibit mycelial growth of *P. plurivora* to 50% (compare Figure 1a and 4a). Similar phosphite concentrations were recorded for *P. plurivora* inoculated and not inoculated plants. No phosphite was detected in roots of control plants.

The *P. plurivora* DNA contents of infected plants increased steadily throughout the experiment and reached the highest values after ten days (Figure 4b). However, the corresponding DNA values of roots of phosphite treated and infected plants were much lower, indicating the powerful action of this compound to protect beech from *P. plurivora*. No DNA of *P. plurivora* was recorded in control and phosphite-treated not-inoculated plants (data not shown).

### *P. plurivora* colonization of plant tissue with or without phosphite treatment

**Figure 5:**
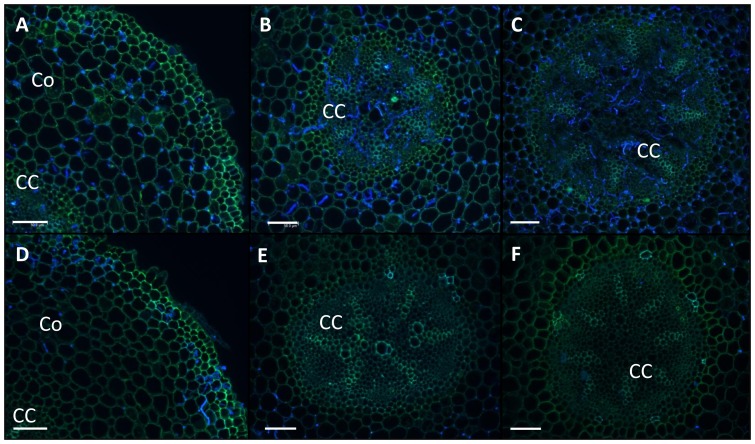
Effect of phosphite treatment on *P. plurivora* colonization of roots. Confocal laser scanning microscopy images of cross-sections of beech sapling roots infected with *P. plurivora* and either treated or not treated with phosphite after 2, 4 and 10 dpi (A, B, C). Beech sapling roots infected with *P. plurivora* and treated with phosphite (0.5%) after 2, 4 and 10 dpi (D, E, F). (Co) cortex, (CC) central cylinder, White bars represent 50 µm.

Two days after infection (dpi) mycelia of the pathogen were already visible throughout the whole cortex tissue (Figure 5a). Four dpi the pathogen had reached the central cylinder and 10 dpi the phloem tissue as well as the pith was severely destroyed by *P. plurivora* (Figure 5b and c). In contrast, in beech plants treated with phosphite, *P. plurivora* mycelia were only visualized in the outer cortex tissue after 2 dpi. However, no mycelia were detected in the central cylinder and the pith even not after 10 dpi at the end of the experiment (Figure 5d to f).

### Expression of defense genes of beech plants treated with or without phosphite and infected with *P. plurivora*

**Figure 6:**
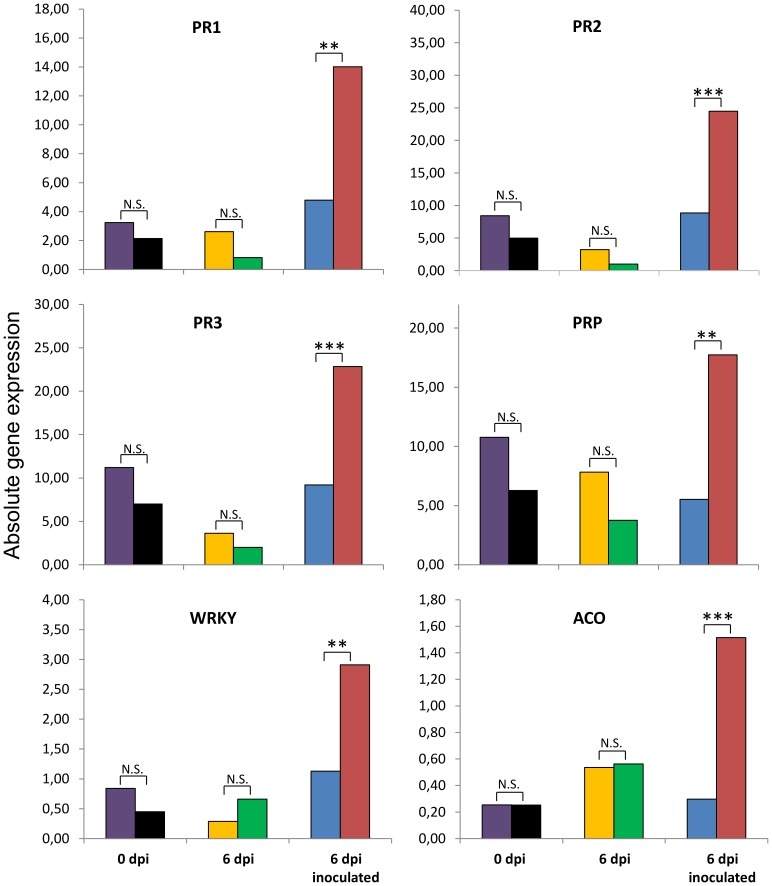
Effect of phosphite treatment and *P. plurivora* infection on the expression of defense-related genes. Absolute gene expression for PR1, PR2, PRP and WRKY (SA signaling pathway) as well as for PR3 and ACO (JA/ET signaling pathway) of beech saplings for all treatments. Purple and yellow bars represent control plants at time zero and after six days. Black and green bars represent phosphite treated plants at time zero and after six days. Blue bars represent infected plants at 6 dpi. Red bars represent phosphite-treated and infected plants at time 6 dpi. Asterisks show level of significance (P≤0.05*; P≤0.01**; P≤0.001***); N.S. Not-significant; n = 4. The experiment was repeated three times showing similar results.

Phosphite treatment did not affect gene expression in beech roots as compared to control plants, neither at time 0 nor at a 6 dpi (purple and black; yellow and green bars, respectively) (Figure 6). Likewise, no statistically significant differences were found when control plants were compared with infected ones at 6 dpi (yellow and blue bars). However, a strong up-regulation for all genes analyzed was measured in plants treated with phosphite and infected with the pathogen at this time point.

## Discussion

In this study, the effects of Phi on beech plants, as well as the ability of the chemical to protect the host towards root infection by the highly aggressive pathogen *P. plurivora* were analyzed. *In vitro* assays proved that mycelial growth was inhibited in a dose-dependent manner and the EC_50_ value was calculated as 34 µg/ml of Phi. Surprisingly, growth of *P. plurivora* was not inhibited totally, even at the highest Phi concentrations, possibly indicating that the *P. plurivora* isolate was able to detoxify phosphite to a certain amount. Similar results were also reported showing that some *P. cinnamomi* isolates were less sensitive to phosphite than others [23]. The authors concluded that sensitivity to Phi may vary within species or even within isolates. Besides affecting mycelia growth, Phi had also an effect on zoospores. Less than 5 µg/ml of Phi was sufficient to stop zoospores production to 50 per cent. Similar EC-values were also calculated for the effect of phosphorous acid, fosetyl-Al, fosetyl-Ca as well as fosetyl-Na on sporangia formation and zoospore release of *P. cinnamomi* and *P. citricola* [18].

It was proved that spraying leaves with 0.5% Phi, four days before roots were challenged with *P. plurivora* zoospores, was sufficient to convert the highly susceptible interaction between beech and *P. plurivora* into a resistant one. No mortality of any seedlings was recorded after 10 days of infection at the end of the experiment. This is in good agreement with the qPCR data for root infection and the laser scanning microscopy images showing the spread of *P. plurivora* in root tissue. Both data sets revealed only a very weak colonization of roots by the pathogen in Phi treated plants as compared to not-treated infected saplings. This strong protective effect of Phi on infected plants could be explained by its concentration in root tissue which was calculated to be about ten times higher than the EC_50_ value. It was also suggested that the degree of resistance of Phi treated host plants correlates with its concentration within plant tissue and that basically the exposure of hyphae to Phi stops the spread of the pathogen [17]. The reason why the Phi concentrations were expressed as µg/ml and not as µg/gDW, as often reported was to make it possible to compare the concentrations of root tissue directly with data calculated for the *in vitro* experiments mentioned above. The Phi data of roots also implicated that the chemical was transported rapidly from leaves into roots. By six days after foliar application the Phi concentrations were as high as at the end of the experiment. These data agree with previous publications showing that Phi was freely translocated in association with photoassimilates throughout the plant in a source-sink relationship and that it accumulated mainly in cell vacuoles [19], [24], [25].

The Phi concentration used in the experiments (0.5%) to induce resistance did not impair physiological parameters of beech saplings, such as net assimilation rate, Jmax, VcMax and water uptake. However, some phytotoxicity was reported for *Eucalyptus marginata* plants when treated with Phi in the rage of 0.25 to 1% [26]. The Phi phytotoxicity may vary within plant species.

Besides local toxicity, restriction of growth of *P. plurivora* could also be explained by direct or indirect stimulation of plant defense responses by Phi. Up to now, the molecular mechanisms underlying Phi-activation of SA or JA/ET signaling pathways are poorly understood. In order to shed light on this, the expression of defense-related genes, which are used as markers for “SA” and “JA/ET” defense pathways, were examined. It was shown by Conrath *et al*. (2002) that up-regulation of defense genes, independent of the signaling pathway, can often be explained by priming. In our experiments, gene up-regulation was only recorded for plants treated with Phi and inoculated with *P. plurivora*, whereas there was no response at all in Phi treated or in inoculated with *P. plurivora* but not treated with Phi. By definition, priming occurs when a plant, prior to exposure by some compound or microorganism, exhibits an augmented defense response under pathogen attack [27]. Comparable to our data, up-regulation of genes of both the SA and the JA/ET signaling pathways after Phi treatment were also found in *A. thaliana* – *P. cinnamomi* interaction [16]. However, in contrast, it was published that Phi effects on Oomycete-challenged plants, were only related with the regulation of genes of the SA signaling pathway [28], [29]. Recently it was shown that there was a transient expression of the 1-aminocyclopropane-1-carboxylic acid (ACC)-oxidase gene in leaves of beech saplings infected with *P. plurivora* on roots [30].

**Figure 7:**
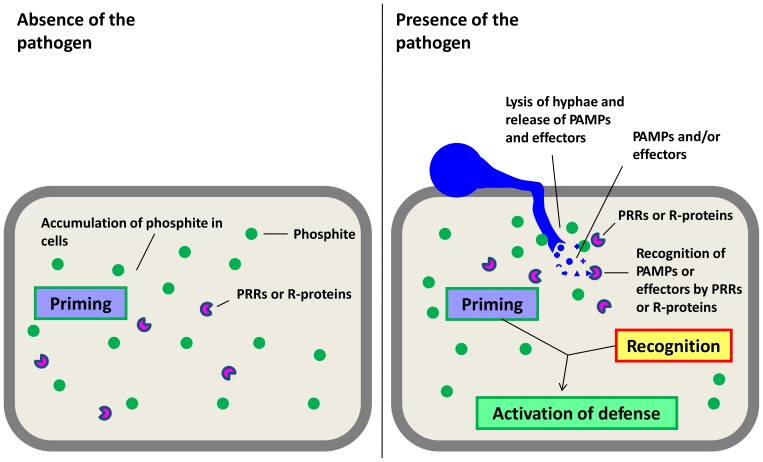
Simplified hypothetical scheme of the possible mode of actions of phosphite on beech saplings infected with *P. plurivora*. After treatment, phosphite accumulates inside cells, priming plants to a subsequent pathogen challenge. During infection phosphite accumulates and will reach toxic concentration inside cells. In consequence the chemical will lyse hypha, which results in the release of PAMPs (pathogen associated molecular patterns) and other effectors. PRRs (pattern-recognition receptors) or R-proteins of the host will recognize these molecules of the pathogen. Finally, together with priming, a fast and strong defense reaction will be activated.

There is still no conclusive explanation regarding the mode of action of Phi and its potential targets in plants. From the results obtained in this study it is possible to conclude that Phi might act in a dual way. In a sub-toxic concentration for the pathogen, the plant might respond via priming, as previously shown [16], [28], [29]. However, if the Phi concentration reaches the toxic threshold inside infected host tissue, *Phytophthora* effectors and/or PAMPs might be released due to hyphae disruption, as recently shown [17], which facilitates pathogen recognition and the burst of defense reactions of the host via SA and JA/ET signaling pathways (Figure 7). Further experiments are necessary to clarify, whether this hypothesis will also prove to be true for other Phi treated host plants susceptible to *Phytophthora* species.

## Materials and Methods

### In vivo

*Phytophthora plurivora* T. Jung and T.I. Burgess, isolate CIT55, which was isolated from a declining beech in Southern Bavaria (Germany), was grown on V8 agar in the dark at 20°C. The strain CIT55 shows sequence identity for the ITS-region as well as the beta-tubuline gene with the type isolate of *P. plurivora* CBS 124093 (ITS: KF990557; b-tub: KF990558).

Radial growth: Petri dishes containing V8 medium with addition of 0, 5, 10, 50 and 100 µg/mL of phosphite (5 plates per Phi concentration) were prepared and one week old *P. plurivora* colonized agar plugs (0.5 cm of diameter) was transferred to the center of the dish. Radial growth of the mycelia was recorded daily for 5 days. The experiment was repeated three times.

Zoospore inhibition: *P. plurivora* was transferred to Petri dishes containing V8 medium. After the colony reached up to 80% of the plates, sporangia development was induced by pouring a solution of 0, 5, 10, 50 and 100 µg/mL of phosphite (5 plates per Phi concentration) on the plate. The solutions were replaced daily for one week. After seven days zoospore release was induced by placing the Petri dishes at 4°C for one hour. The amount of zoospores released was recorded using a Thoma chamber. The experiment was repeated three times.

### In planta

Plant growth conditions: Seeds of European beech (*Fagus sylvatica L*.) were germinated and grown in root trainers with sterile vermiculite for 3 months at 20°C and light conditions of 250 µmol m^−2^ s^−1^ photosynthetic photon flux density (PPFD). Three days before initiating the experiment, the seedlings were carefully removed from the containers. The roots were rinsed of the substrate and placed in 50 mL Falcon tubes containing 50 mL of distilled water and sealed with Parafilm.

Phosphite treatment: Plants were treated with phosphite 4 days before inoculation with *P. plurivora* zoospores (T = −4). The leaves were sprayed until run-off with potassium-phosphite using a plastic spray bottle. The phosphite solution was prepared by mixing commercial KOH with H_3_PO_3_ (both from Sigma-Aldrich Chemie GmbH, Steinheim, Germany) at pH 3.0, with a final concentration of 0.5%. Roots were inoculated as follows: zoospores (5×10^5^ zoospores per plant) were carefully pipetted from the zoospores suspension and transferred to the water surroundings the roots inside the falcon tubes.

Experimental design and inoculation of plants with zoospores: Four months old *F. sylvatica* seedlings were treated as follows:(a) control: not Phi-treated/not-inoculated (Con); (b) phosphite treated/not-inoculated (Phi); (c) not phosphite-treated/*P. plurivora* inoculated (Plu); (d) phosphite treated/inoculated with *P. plurivora* (Phi-Plu). Six plants were used for each treatment. The experiment was repeated three times.

### Quantification of phosphite in roots

For the determination of phosphite within different plant organs, beech seedlings were harvested, washed with deionized water, dried with cellulose tissue, separated into leaves, stems and roots and freeze dried for 24 hours. The fresh and dry weight of each organ was determined before and after freeze drying. All samples were ground to a fine powder using a ball mill and phosphite was extracted from 50 mg of powdered plant tissue in 500 µL HPLC-water [31]. The samples were vortexed vigorously and incubated over night at room temperature (RT) in the rotary overhead-shaker. Afterwards samples were centrifuged for 10 minutes at 15,000 g. The supernatant was filtered through a 0.45 µm nylon filter and stored at −20°C until analysis. Ion chromatography of phosphite (H_2_PO_3_^−^) was performed using 20 mM succinic acid as the mobile phase at a flow rate of 0.8 mL per minute [32]. The HPCL system consisted of a L-6200 A Pump (Hitachi), a Vidac 302 Anion Column (250×2.1 mm; 10 µm, Grace) heated at 40°C in a HIC-6 A Column oven (Shimadzu), and of a CDD-6 A conductivity detector (Shimadzu) heated to 43°C.

### Disease assessment and plant physiology

The plants were monitored daily for root necrosis, growth of visible mycelia on the root surface, wilting of leaves and mortality.

Gas exchange measurements were conducted at 0, 2, 4,6 and 8 dpi using a CO_2_/H_2_O diffusion porometer equipped with a broad-leaf LED cuvette (LI-6400, LI-COR, Lincoln, Nebraska, USA). All the measurements were conducted under steady-state conditions of 23°C (leaf temperature), between 50% and 60% relative humidity and 400 ppm CO_2_ concentration (in the reference air), 250 µmol m^−2^ s^−1^ PPFD, and 500 mL min^−1^ air flow.

To analyze the activity of the enzyme RuBisCO, an A/C_i_-Curve was performed at 8 dpi with the LI-COR LI6400 using the default program of the device. Leaf CO_2_ uptake (A) versus intercellular CO_2_ concentration (C_i_) curves provide information about the limitation of photosynthesis.

The slope of an A/C_i_-curve was used to calculate RuBisCO activity (V_cmax_), the maximum rate of electron transport (J_max_) and mitochondrial respiration (R_d_). The data were analyzed with a Microsoft Excel based macro (http://landflux.org/Tools.php) on the models of [33]–[35].

### qRT-PCR and gene expression

Genomic DNA was extracted from 20 mg freeze-dried and milled root material using the DNeasy plant mini kit (Qiagen, Hilden, Germany) and was further purified using the Wizard® DNA clean up system (Promega, Mannheim, Germany) according to the manufacturer's protocols. The DNA was diluted 1∶10 in H_2_O to prevent the inhibition of the PCR reaction. The amount of *P. plurivora* DNA in 5 µL of root extract was determined by TaqMan quantitative PCR using an SDS7700 sequence detection system (Applied Biosystems, Germany), with the primer pair P5/P6 and the fluorogenic probe F3 labelled with FAM as a reporter dye and TAMRA as a quencher. All of the analyses were performed in three technical repetitions using ABsolute QPCR ROX chemicals (ABgene, Hamburg, Germany) and performing 40 cycles of denaturation at 95°C for 15 s and annealing/extension at 62°C for 60 s. The Ct values of the samples were compared with a standard curve that was generated for pure *P. plurivora* genomic DNA extracted from mycelia grown in liquid culture (mineral medium M1), supplemented with glucose 10 g/L and L-asparagine 2 g/L). The standard curve concentrations ranged from 1 pg DNA mL^−1^ to 10 ng DNA mL^−1^ in five steps.

For the extraction of total RNA, 50 mg of roots were ground in liquid nitrogen using a mortar and pestle. The total RNA was extracted using the MasterPure Plant RNA Purification kit (Epicentre Biotechnologies, Madison, WI, USA) according to the manufacturer's protocol, including a DNAse I treatment. The concentration and quality of extracted RNA was measured using a BioMate 3 Photometer (Thermo Fisher Scientific, Ulm, Germany). cDNA was reverse transcribed using 1 µg of total RNA with oligo-dT primers and the MMLV Reverse Transcriptase 1st strand cDNA Synthesis kit (Epicentre Biotechnologies, Madison, WI, USA). The transcript levels of specific genes were analysed by using 0.05 µg of cDNA by qRT-PCR in three technical replicates using the ABsolute SYBRGreen ROX chemicals (ABgene, Hamburg, Germany) and performing 40 cycles of denaturation at 94°C for 30 s, annealing at 60°C for 30 s, and extension at 72°C for 30 s [36].

In order to shed light on Phi-induced gene defense responses and to elucidate whether these responses are mediated via SA or JA/ET signal transduction, transcriptional analysis of defense genes relating to both pathways was performed. The induction of defense related genes by Phi without inoculation at 0 dpi and 6 dpi (which are 4 and 10 days after Phi treatment, respectively) and after *P. plurivora* inoculation (6 dpi, which is 10 days after Phi treatment) was examined. The transcript level of defense genes of the SA (PR1, PR2, PRP and WRKY) and JA/ET (PR3, and ACO) pathways was quantified using real-time reverse-transcription polymerase chain reaction (qRT-PCR). The relative expression levels were calculated comparing Phi treated and inoculated plants with not infected plants using the Relative Expression Software Tool REST 2009 (Qiagen, Hilden, Germany)[37]. Actin, tubulin and GAPDH were used as reference genes. A list of primers and sequences used in this study can be found in the Table 2.

**Table 2:** Oligonucleotides used within this study.

name	primer	primer sequence 5′ – 3′
*P. plurivora* ITS1	P5	TCAACCCTTTTAGTTGGGGGTC
*P. plurivora* ITS1	P6	TTTAAAACAAAAAGCTACTAGCCCAGAC
*P. plurivora* ITS1	F3	FAM-CTTTTTTTGCGAGCCCTATCATGGCGA-TAMRA
Actin	Actin-Fw	AGAGATTCCGTTGCCCAGAA
	Actin-Rv	TGGATTCCAGCAGCTTCCA
PR1	PR1-Fw	CACTGTGATTGAGGGTGATG
	PR1-Rv	GCTCTTCAACACAGATCCTC
PR2	PR2-Fw	TCAAAGGGGGTACACCAAAG
	PR2-Rv	TCARCAGTGACATCCCATAGTC
PR3	PR3-Fw	GGTGGAAGATCGCATTGGGTTC
	PR3-Rv	CACAAGACTACAAGGTCAGGCATCC
PRP	PRP-Fw	GGTTTCAAGAGGAAAAAGTGCCAGT
	PRP-Rv	GCTTGCCATCCAGGTTTGTTC
ACO	ACO-Fw	CTGGTGGGATCATCTTACTC
	ACO-Rv	CAATAGAATGGCGCATAGGG
WRKY	WRKY-Fw	TTTCTCACTGGACACGCTGG
	WRKY-Rv	GATGGCTACCGTTGGAGGAA
Tubulin	Tubulin-Fw	TGAGTTGCTCAGGGTGGAAAA
	Tubulin-Rv	CGAGCCCACTGTCATCGAT
GADPH	GADPH-Fw	GATAGATTTGGAATTGTTGAGG
	GADPH-Rv	AAGCAATTCCAGCCTTGG

### Confocal laser-scanning microscopy

To prepare the samples after harvest, the root material was fixed in phosphate-buffered saline (PBS) (pH 7.2) with 3% formaldehyde. The root samples were manually sliced using a razor blade, and the section were washed in PBS/0.2% Tween three times. The root sections were then blocked for 30 min with 100 mM glycine in PBS/0.2% Tween. After washing the material again, a protein block was performed with 1% BSA in PBS (pH 7.2) for 30 minutes.

The root sections were then incubated for 2 h at 37°C with the primary antisera, the commercial antibody against *P. plurivora* (rabbit anti-*Phytophthora* spp. polyclonal antiserum from Loewe Diagnostica®, at 1∶400). After incubation, serial washings (two x 10 minutes) were performed with PBS/0.2%Tween and then with PBS.

The root sections were incubated for 60 minutes at 37°C with secondary antisera (goat anti-rabbit conjugated to Pacific Blue, Invitrogen®, at 1∶200). Before confocal laser-scanning microscopy, the samples were washed several times with PBS/0.2% Tween and with PBS (pH 7.2).

The confocal imaging was performed using a Leica TCS SP5 confocal laser-scanning microscope (Leica Microsystems CMS GmbH, Mannheim, Germany). Pacific Blue was excited at 405 nm and detected between 430 and 480 nm. Plant auto-fluorescence was detected between 500 and 550 nm after excitation with a 488 nm laser-line.

### Statistics

The data were analysed using the Statistics software SPSS. A time series factorial was used to analyse the interaction of phosphite and *P. plurivora* over time. In case of no interaction an ANOVA One-Way was conducted to compare data at each time point with 5% significance (P≥0.05).
